# Supplementary material for: Antiviral Efficacy of RNase H-Dependent Gapmer Antisense Oligonucleotides against Japanese Encephalitis Virus
Source: Int J Mol Sci. 2023 Oct 2;24(19):14846. doi: 10.3390/ijms241914846 (PMC10573891; doi:10.3390/ijms241914846)
Supplement: Supplementary file 1 [file ijms-24-14846-s001.zip › ijms-2579899-supplementary.docx]

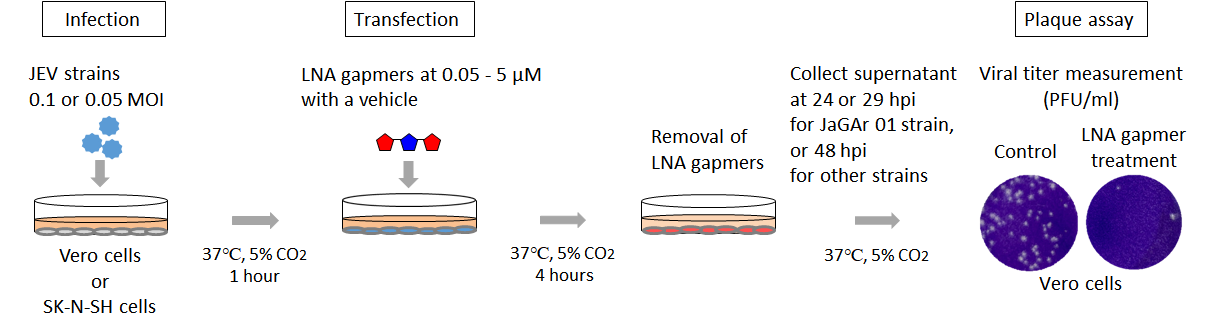


**Figure S1. Experimental scheme of LNA gapmer transfection in JEV-infected cells.** Vero or SK-N-SH cells infected with the JEV strains at 0.1 or 0.05 MOI were transfected with LNA gapmers and a vehicle, Lipofectamine RNAiMAX. At 24 or 29 hours post-infection (hpi) of JaGAr 01, the supernatants were collected for the viral titer measurement by plaque assay using Vero cells. The supernatants of SK-N-SH cells infected with JEV wild-type strains were harvested at 48-hpi.


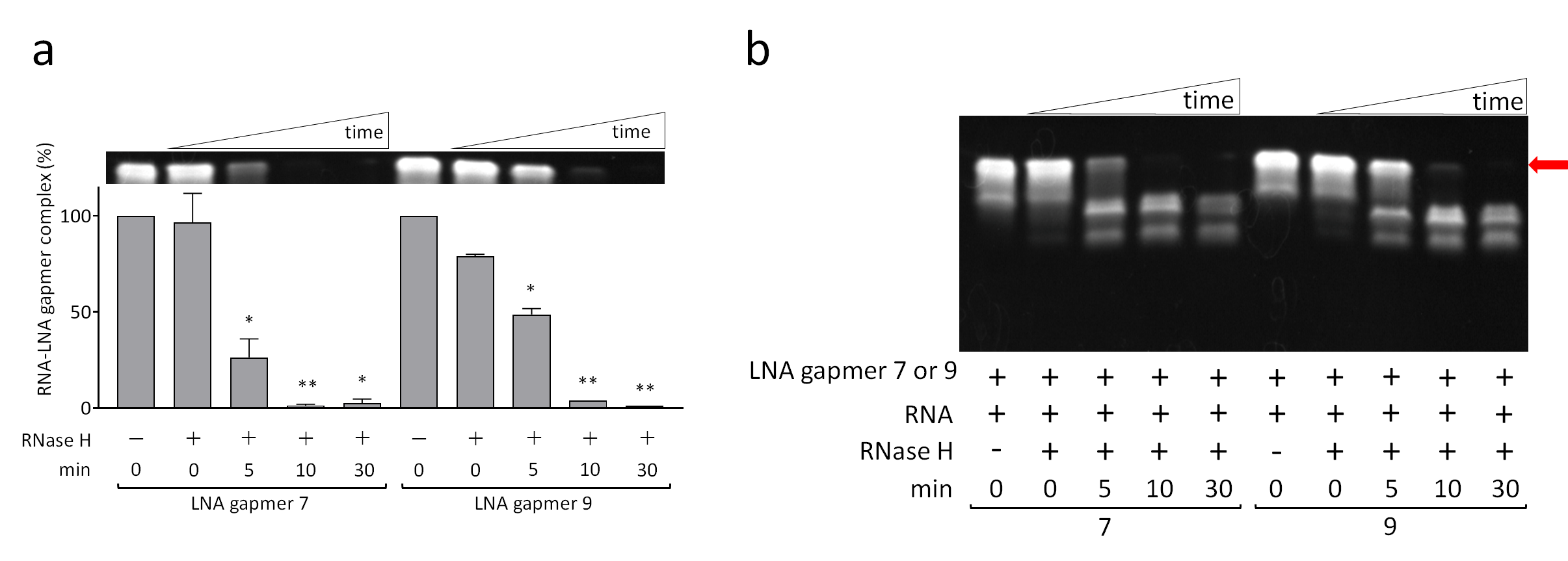


**Figure S2. RNase H-mediated degradation of synthetic JEV RNA bound to LNA gapmers 7 and 9 over time as represented by RNA cleavage assay.** (**a**) The representative band image of the synthetic JEV RNA and LNA gapmer complex are shown above the graph. The graph calculated with band intensities represents the mean and SD of three independent experiments. Shirley-Williams test was performed for statistical analysis: * *p* < 0.05, ** *p* < 0.01 versus 0 min just after the RNase H addition. (**b**) An uncropped image of Figure S2a that shows the degradation of synthesized RNA by RNase H over time up to 30 min. A red arrow indicates the JEV RNA and LNA gapmer complex.


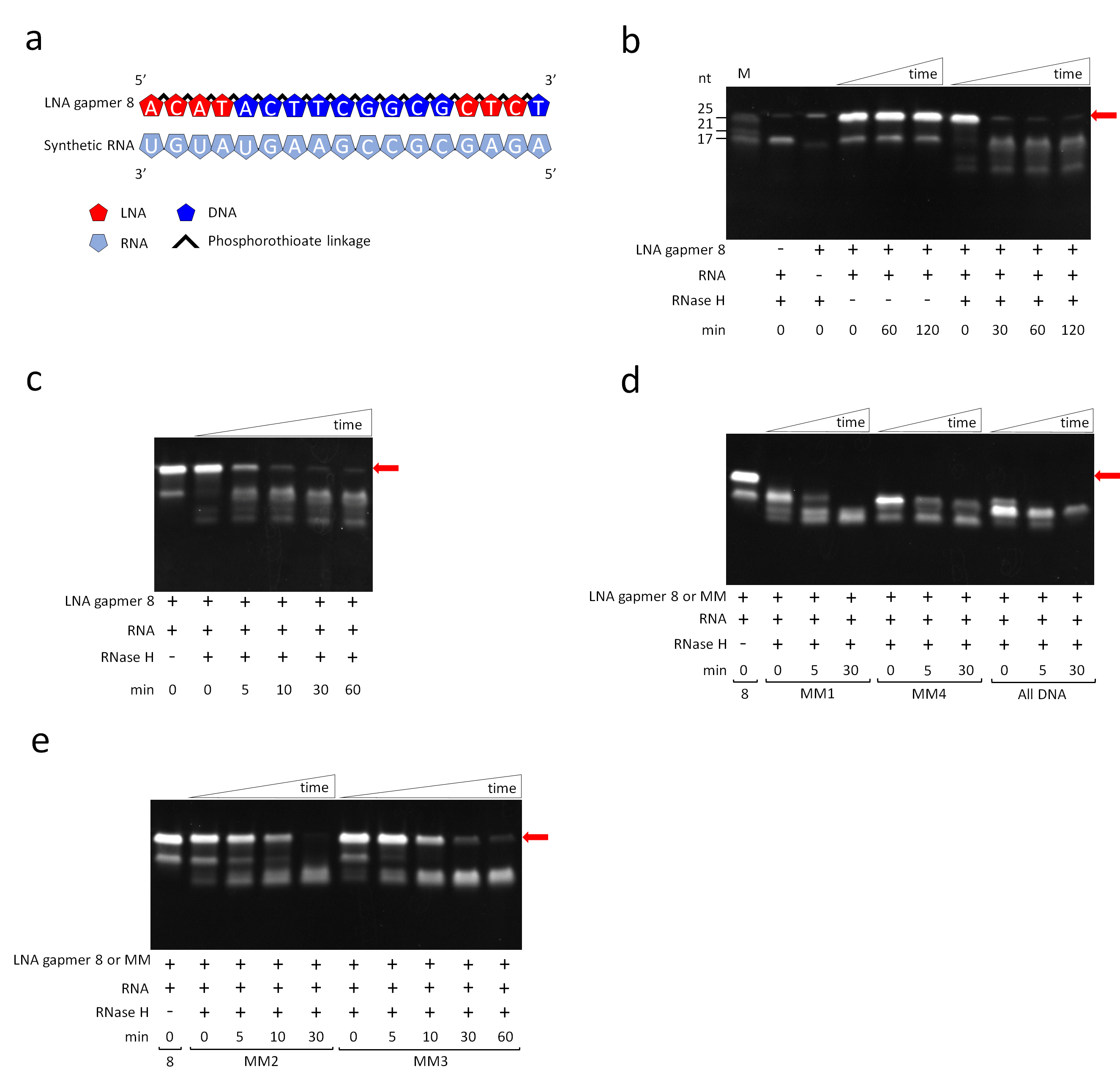


**Figure S3. RNA cleavage assay using the original LNA gapmer 8 and mismatched LNA gapmers.** (**a**) A schematic of the hybridization of LNA gapmer 8 and the synthetic JEV RNA. (**b**) LNA gapmer 8-mediated degradation of the synthetic JEV RNA in the presence or absence of RNase H. A representative image is shown. M, RNA marker; red arrow, RNA and LNA gapmer complex. (**c**) An uncropped original image of Figure 3c with the RNA degradation by LNA gapmer 8 with time. (**d**,**e**) Uncropped images of Figures 3d and e, respectively, in RNA cleavage assay using mismatched LNA gapmers and ASO composed of only DNA.


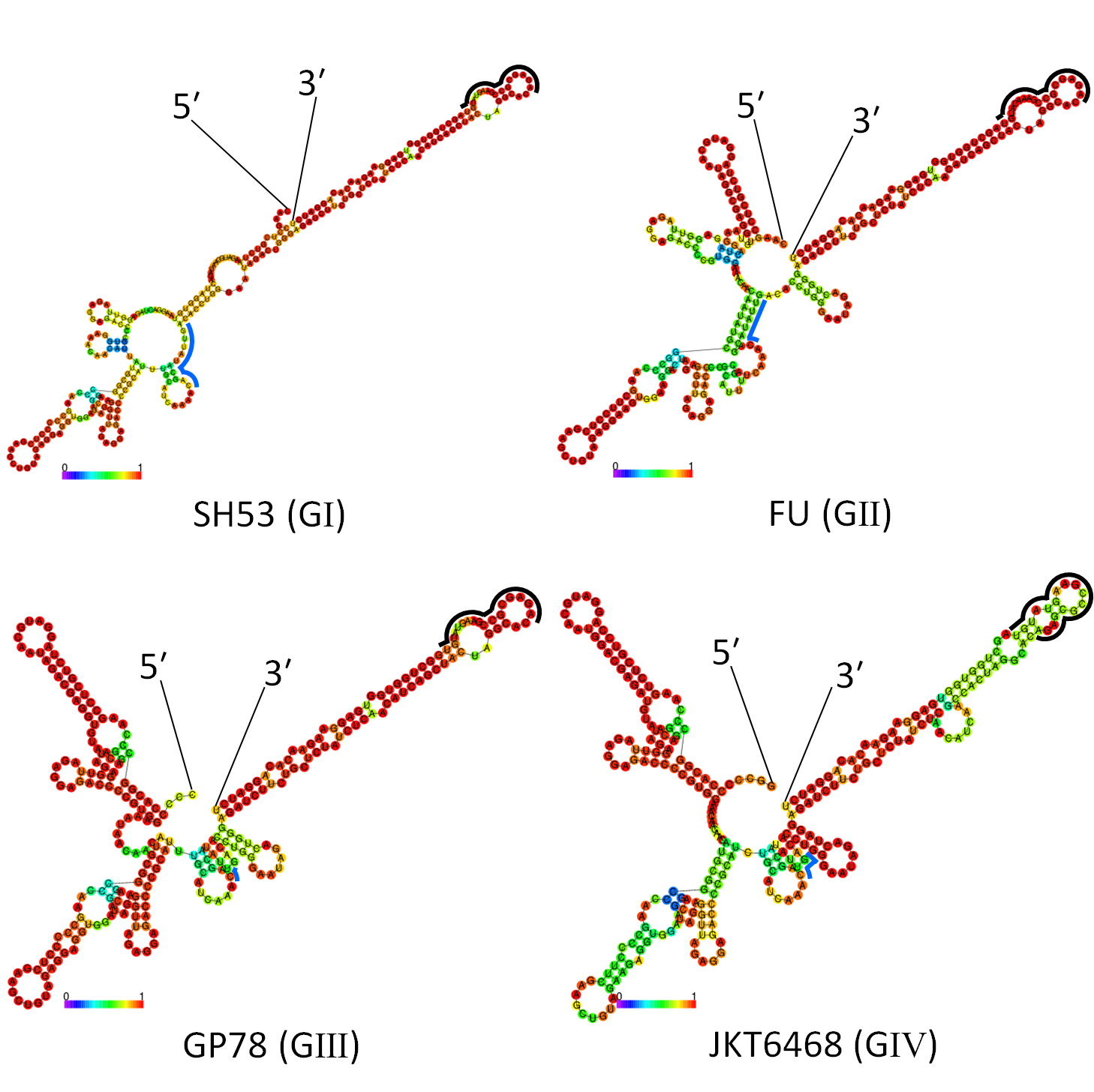

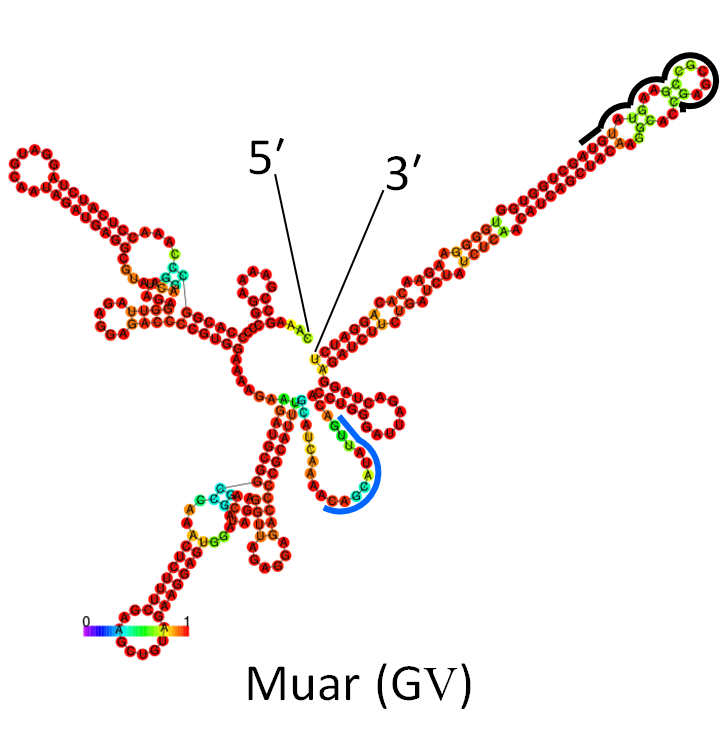


**Figure S4. The 3’ UTR stem-loop RNA secondary structures in the five JEV genotypes.** Predicted by RNA fold program, the 3’ UTR secondary structures encoded in 10,700-10,977 nt of the following strains were shown as representatives of individual JEV genotypes: SH53 (GI, accession no. JN381850.1), FU (GII, AF217620.1), GP78 (GIII, AF075723.1), JKT6468 (GIV, AY184212.1), and Muar strain (GV, HM596272.1). Black lines indicate the stem-loop regions targeted by LNA gapmers developed in this study. Blue lines indicate the 3’ UTR CS Ⅰ region. A heat map color represents the base-pairing probabilities. The redder they are, the more likely they are to form the indicated secondary structures.

**Table S1. Classification of RNAs with two mismatches or gaps complementary to LNA gapmers as detected by GGGenome.**

|  | **The number of complementary RNAs with two mismatches or gaps** | | | |
| --- | --- | --- | --- | --- |
|  | **mRNA** | **non-coding RNA** | **miscellaneous RNA** | **Total** |
| LNA gapmers 7 and 8 | 4 | 5 | 1 | 10 |
| LNA gapmer 9 | 57 | 17 | 7 | 81 |
